# Postoperative Epidural Analgesia in Cesarean Section: Comparison of Therapeutic Schemes

**DOI:** 10.7759/cureus.12166

**Published:** 2020-12-19

**Authors:** Raquel Fonseca, Décia Gonçalves, Sónia Bento, Elisabete Valente

**Affiliations:** 1 Anesthesiology, Leiria Hospital Center, Leiria, PRT

**Keywords:** postoperative pain, chronic and acute pain management, cesarean birth, local analgesia

## Abstract

Background

Cesarean section is associated with moderate to severe postoperative pain. Its adequate control is fundamental to postpartum functional recovery, prevention of chronic pain, and postpartum depression. In this context, neuraxial analgesia has shown superior results. However, the best pharmacological regimen is still unknown. This study intended to compare the performance of three epidural therapeutic schemes (0.1% ropivacaine combined with epidural morphine vs 0.2% ropivacaine combined with epidural morphine vs morphine bolus) in pain intensity and its adverse effects in the early postoperative period of cesarean section.

Methods

A retrospective observational study was carried out. The sample included 204 women who underwent cesarean section after previous epidural catheter placement. Demographic and clinical data were collected. Pain intensity in rest, movement at 24 and 48 hours, and adverse effects (pruritus, nausea, sedation, respiratory depression, hypotension, urinary retention and paresthesias) were recorded.

Results

Statistical analysis revealed no differences in mean pain scores between groups on the first and second postoperative days. The incidence of adverse effects was significantly lower in the morphine bolus group.

Conclusion

Epidural morphine therapy is an effective option with an adequate safety profile. The addition of a local anesthetic seems to offer no benefit in this context, increasing the incidence of adverse effects.

## Introduction

The prevalence of cesarean sections remains above the desirable, representing a rising value of 21.1 % of all deliveries in 2015 [1]. It is a surgical procedure unequivocally associated with postoperative pain, classified as moderate to severe by 51% of women [2].

Adequate control of acute postoperative pain is crucial in reducing the incidence of chronic postoperative pain [3]. Regarding cesarean section, effective analgesia has additional benefits, particularly in improving functional recovery that favors early interaction between mother and baby. It also reduces the risk of postpartum depression [4,5].

The most recent guidelines recommend a neuraxial anesthetic approach over general anesthesia in cesarean section. Similarly, they suggest analgesia with spinal epidural opioids, as it has superior results when compared to systemic therapy [6].

The timing (elective, urgent or emergent) and previous placement of epidural catheter to labor analgesia are crucial to the anesthetic and analgesic strategy.

While some studies suggest superiority of intrathecal morphine in postoperative analgesia in elective cesarean section [7], the best analgesic option when a functioning epidural catheter is already in place for labor analgesia is still unclear. The latter scenario is frequent in urgent cesarean section.

Huang et al. (2015) compared *de novo* subarachnoid spinal block (SB) to epidural in cesarean section in patients previously submitted to epidural labor analgesia. Although total anesthetic time was lower, there was a significantly higher incidence of hypotension and a trend towards higher incidence of technical failure in the SB group [8]. However, postoperative analgesic therapy was not studied.

In patients exhibiting previous epidural catheter, alternative analgesic schemes are diverse: epidural analgesia with continuous opioid and local anesthetic (LA) perfusion or Patient Controlled Epidural Analgesia (PCEA), LA perfusion and programmed opioid epidural bolus.

Among LA, ropivacaine showed superior safety profile concerning cardiotoxicity and risk of motor block [9]. Morphine, due to its water solubility, offers prolonged postoperative analgesia, between 18 and 24 hours, making it the most frequent choice in this context. It is, however, related with multiples and dose-related adverse effects: pruritus; nausea and vomiting; and urinary retention [10].

Previous studies regarding patients undergoing abdominal surgery show a potential synergistic effect of local anesthetics, when combined with epidural opioid [11]. Moreover, this combination proved to be opioid-sparing, increasing overall security. This analgesic scheme lacks comparative efficacy studies with morphine bolus administration in the context of cesarean section.

Thus, in this study, we aimed to compare epidural therapeutic schemes (ropivacaine and morphine vs morphine bolus) in pain intensity and adverse effects, in early post-cesarean section period.

## Materials and methods

Sampling

We carried out a retrospective observational study, comprising a review of data relating to all cesarean sections elapsed in the period between January 2018 and December 2018. This study was approved by the ethics commission of Centro Hospitalar de Leiria.

Based on this sample, we selected all cases in which epidural analgesia was performed.

Daily evaluation parameters of acute postoperative pain were recorded, according to the institutional protocol, namely: (1) pain intensity (numerical rating scale - NRS) at rest and movement; (2) incidence of adverse effects (pruritus, nausea, sedation, respiratory depression, hypotension, urinary retention and paresthesia/bromage); and (3) therapeutic attitude. All unexpected occurrences were also recorded.

Exclusion criteria included other therapeutic schemes (exclusive intravenous analgesia, *transversus abdominis* plane block or intrathecal morphine) and absent or incomplete postoperative evaluation records. 

**Figure 1:**
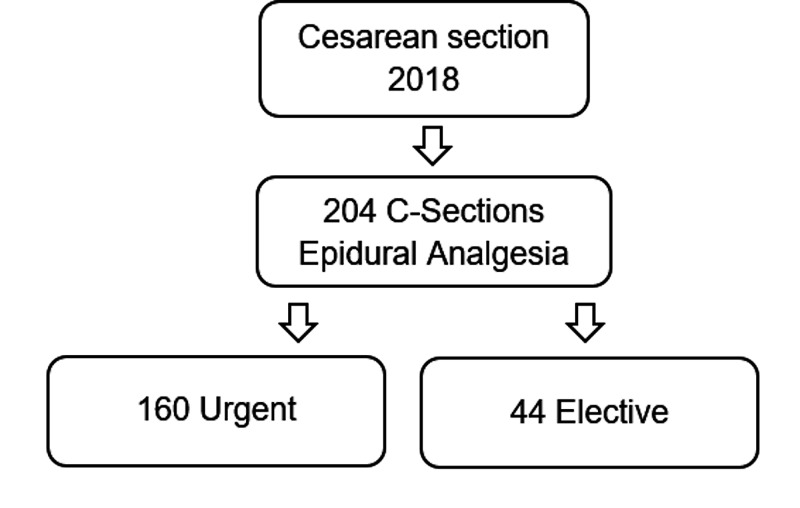
Sampling

A total of 204 cases were obtained (N = 204) (Figure 1).

Of these, 160 corresponded to urgent cesarean sections, in which the epidural catheter had been previously placed for labor analgesia, and its functionality was assured. The remaining 44 were elective cesarean sections under combined spinal-epidural anesthesia in which levobupivacaine 5 mg/ml (10 mg) and sufentanil 5 mcg/ml (2.5 mcg) were administered intrathecally. According to the lipophilic profile of sufentanil, a hypothetical contribution to the management of acute postoperative pain was neglected. All patients maintained bladder catheterization for the first 12 hours.

The therapeutic prescription always included complementary analgesia -paracetamol 1G 8/8 hours, diclofenac 75 mg 12/12h and tramadol 100mg ev if relapsing pain - and a rescue antiemetic.

Demographic and clinical data such as age, comorbidities, previous pregnancies and gestational age at labor were also collected.

Statistical analysis

Acquired data were analyzed using Statistical  Package for Social Science 25.0 (SPSS) software (IBM Corp., Armonk, NY). A descriptive analysis was carried out, with results expressed in relative frequencies (%) and measures of central tendency (mean) and dispersion (minimum, maximum and standard deviation). We used analysis of variance (ANOVA) to assess potential differences between groups, applying Pearson Chi-square Test for categorical variables. Games-Hollew *post-hoc* test was used when stratified comparison between groups was needed. Spearman correlation coefficient was used chosen for correlational analysis. Confidence intervals of 95% and a significance level of .05 were applied.

## Results

The sample consisted of 204 women, aged 18 to 49 years (mean = 33.3; SD = 5.8). Table 1 shows the characterization of the participants regarding age, medical history, number of pregnancies, gestational age (term/preterm) and timing of cesarean section (urgent/elective). The most frequent comorbidities were pre-existing or gestational hypertension (n = 13, 6.4%), obesity (n = 13, 6.4%), gestational diabetes (n = 13, 6.4%) and thyroid disease (n = 10, 4.9%). There were no statistically significant differences between the analgesic regimen groups regarding demographic and clinical data.

**Table 1:** Sample characterization. ^a.^Mean (standard deviation); ^b^minimum-maximum; ^c^n (%); ^d^T test; ^e^Chi-square test.

	Total, n = 204	Urgent, n = 160	Elective, n =44	p
Age (years)^a,b^	33.3 (5.8) 18-49	33.0 (5.6) 18-46	34.3 (6.3) 21-49	0.186^d^
Number of pregnancies ^a,b^	1.9 (1.1) 1-6	2.5 (1.3) 1-6	1.1 (0.3) 1-6	0.001^d^
Gestational Age^c^				
Preterm	14 (6.9)	11 (6.9)	3 (6.8)	
Term	190 (93.1)	149 (93.1)	41 (93.2)	0.989^e ^
Comorbidities^c^				
Yes	86 (57.8)	60 (37.5)	26 (59.1)	0.010^e^
No	118 (42.2)	100 (62.5)	18 (40.9)	

**Figure 2:**
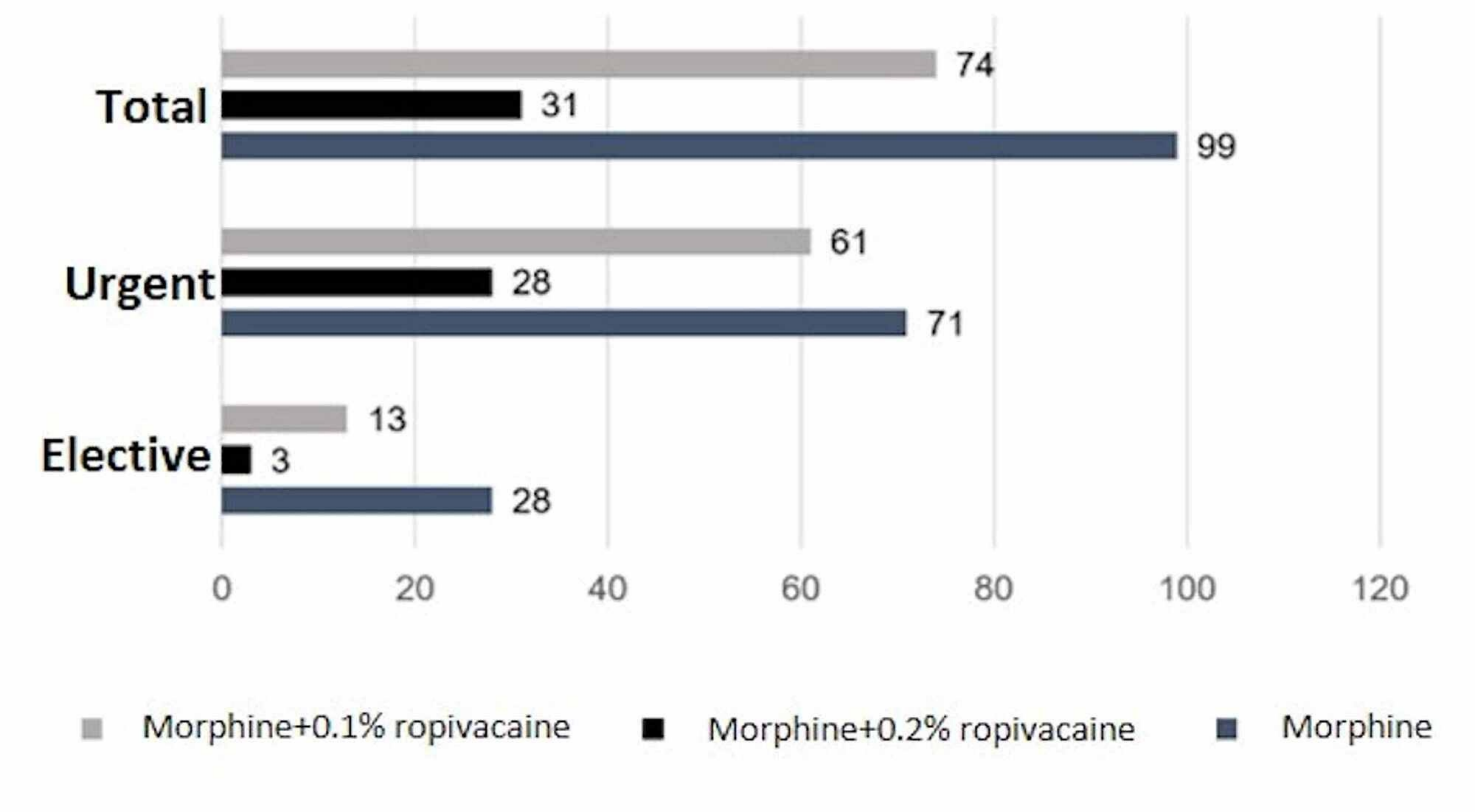
Analgesic scheme according to type of surgery

Three epidural regimens were evaluated: 0.1% ropivacaine + morphine (RM1) or 0.2% ropivacaine + morphine (RM2) on continuous administration by 100 mL Drug Infusion by Balloon (DIB) device (to be infused over 24 hours) and morphine in programmed scheme bolus (M). The most frequently used analgesic regimen was the programmed morphine bolus (Figure 2), within 12/12 hours, with doses between 2 mg and 6 mg in 24 hours.

The mean daily dose of morphine was significantly lower (p<.001) among patients in the RM1 (= 4.0) and RM2 (= 3.9) and M (= 5.3) groups and similar between RM1 and RM2 (p > 0.05).  The mean intensity of postoperative pain at 24 hours, assessed by the NRS, was mild at rest and moderate in movement in all groups. At 48 hours postoperatively, the pain was slight, even in motion.

There was no significant correlation between number of pregnancies and pain intensity in motion at 24 hours (p = 0.984; ρ = -0.001) or 48 hours (p = 0.652; ρ = -0.652). The mean intensity of pain on the move was similar at 24 [t (202) = 0.413; p > 0.05] and 48 hours [(t (141) = .483; p > 0.05], regardless of cesarean section timing.

Statistical analysis revealed no differences in mean pain scores between RM1, RM2 and M groups on the first and second postoperative days (Table 2).

**Table 2:** Postoperative pain (day 1 and day 2). ^a^Mean (standard deviation); ^b^ANOVA.

	Rest pain Day 1 (EN)^a^	p^b^	Movement-Evoked Pain Day 1 (EN)^a^	p^b^	Rest pain Day 2 (EN)^ a^	p^b^	Movement-Evoked Pain Day 2 (EN)^a^	p^b^
Total (n = 204)	0.9 (1.6)	0.446	4.0 (2.2)	0.692	0.7 (1.1)	0.697	3.0 (2.0)	0.732
M group (n = 99)	0.8 (1.5)		4.1 (2.3)		0.7 (1.2)		2.9 (2.0)	
RM2 group (n = 31)	1.0 (1.6)		3.8 (2.2)		0.5 (0.8)		2.8 (2.0)	
RM 1 group (N = 74)	1.1 (1.6)		3.8 (2.1)		0.7 (1.2)		3.1 (2.0)	

**Figure 3:**
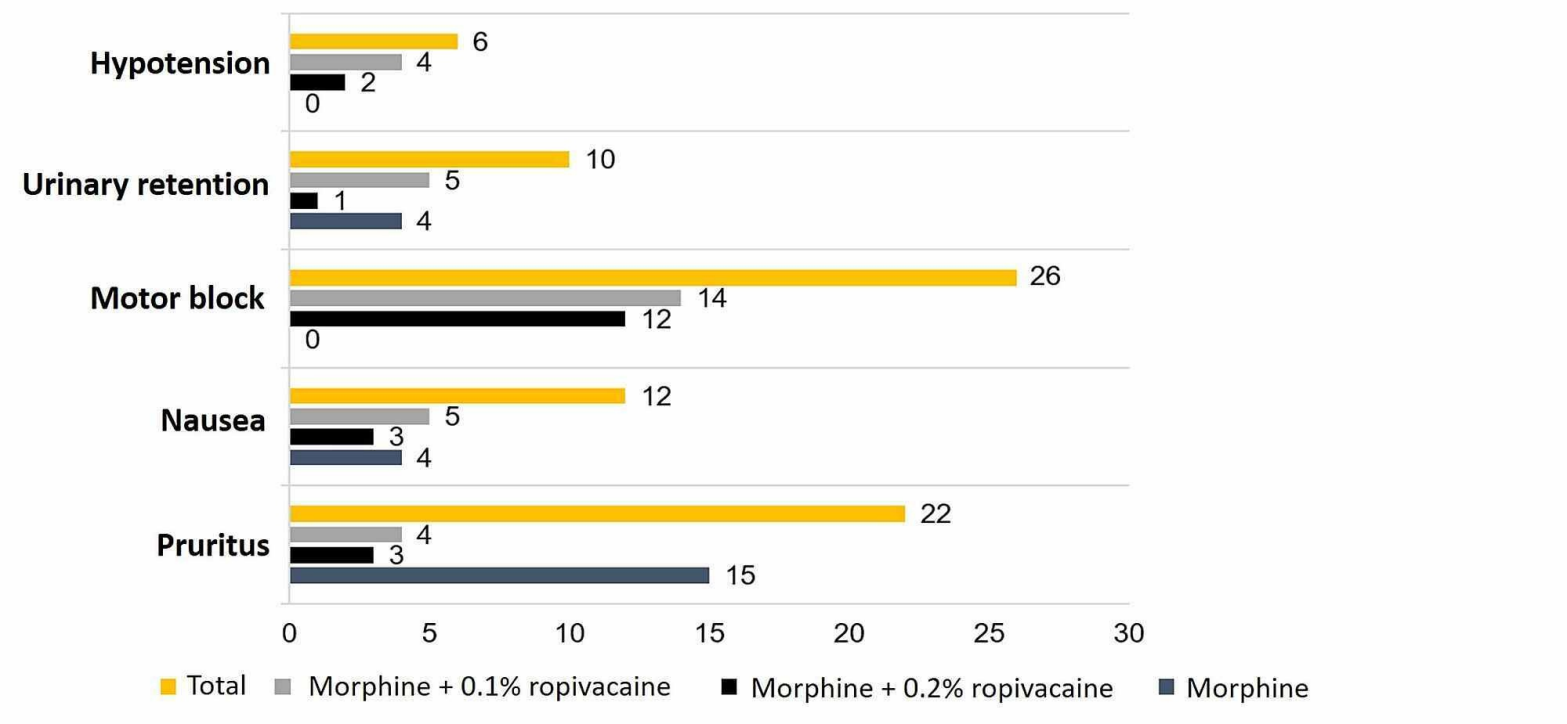
Adverse effects (n).

The most frequent adverse effects were motor block (n = 26; 12.7%) and pruritus (n = 22; 10.8%). In the sample studied there was no respiratory depression or sedation. The incidence of adverse effects was distinct among the three groups, significantly lower in M group [χ2 (3) = 12.3; p < 0.001]. Considering the adverse events separately, a statistically significant difference was observed in the occurrence of motor block, predictably higher in groups RM1 and RM2 [χ2 (2) = 32.5; p = 0.000], and in RM2 when compared to RM1 [χ2 (1) = 4.6; p = 0.032] (Figure 3).

Although tending to be more frequent in group M, the different incidence of pruritus did not reach statistical significance [χ2 (2) = 3.8; p > 0.05]. However, among patients who experienced itching, morphine dose was significantly higher [(t(28.1) = -1.8; p = 0.050].

## Discussion

Neuraxial analgesia techniques have assumed a role of increasing importance in obstetrics, with satisfactory results in pain control. Additionally, they obviate many of the undesirable effects of systemic therapy, in particular in breastfeeding concerns, *ileus* and prolonged immobilization [12].

Our results show that epidural analgesia with LA and morphine also reduces post-cesarean pain to mild to moderate intensity when compared with the singular use of morphine.

Previous research lines have studied the analgesic efficacy of LA, lipophilic opioids and morphine, isolated and in different combinations [13-17]. Despite the heterogeneous results, ropivacaine systematically proved to have superior safety profile and morphine prevailed as the most effective and long-lasting opioid in this context. However, to our knowledge, our investigation work is the first to comparatively analyze the performance of morphine with continuous infusion ropivacaine, combining the benefits found for both drugs, and morphine alone.

The aim of this study was to compare the performance of analgesic regimens including LA and morphine versus morphine administered epidurally in acute postoperative cesarean section pain. Results suggest no superiority in the simultaneous administration of ropivacaine, as pain intensity was similar in both therapeutic schemes.

Although LA therapy allowed the use of a lower dose of morphine, that reduction did not translate into a safer adverse effect profile. In fact, the incidence of adverse effects was significantly lower in group M, largely due to motor blockade/paresthesias in groups RM1 and RM2.

However, the interpretation of these results should be cautious, considering the low incidence of adverse effects on the overall sample. In fact, as objectified in the correlational study, a higher dose of morphine will tend to translate into more adverse effects such as pruritus, which might remain undisclosed in this sample size. On the other hand, as suggested by Chen et al. (2011), the occurrence of motor block, not always means delayed ambulation, so that motor blockade in RM1 and RM2 groups has an uncertain clinical impact and may not deserve such a penalizing appreciation [13].

However, the primary conclusion is that daily mean epidural morphine doses of 5 mg, similar to those used in group M, may be a safe and no inferior option to simultaneous LA therapy in postoperative cesarean section analgesia.

The assessment of patient satisfaction with the established therapy may be a decisive element in the final result of the comparative analysis of these schemes. In fact, despite apparent equity in analgesia results, continuous infusion devices such as DIB or PCEA may be considered uncomfortable by postpartum women, resulting in a lower overall satisfaction rate comparing to manual bolus administration without accessory devices.

Also, costs associated with them should also be taken into account. Vercauteran, in a cost-effectiveness study, demonstrated a higher expense of 33 euros when using Patient-Controlled Analgesia (PCA) devices [18]. Similarly, device-free epidural administration could be an added advantage.

Subsequent studies weighing these factors - clinical, economic, and patient-associated - may lead to more reliable and reproducible conclusions about postoperative cesarean section analgesia.

## Conclusions

Cesarean section is associated with moderate to severe postoperative pain whose control is key to postpartum functional recovery and prevention of chronic pain. While some studies suggest superiority of intrathecal morphine in postoperative analgesia in elective cesarean section, the best analgesic option when a functioning epidural catheter is already in place for labor analgesia is still unclear. In this study, no benefit in postoperative pain was found when morphine was added to epidural LA. Interestingly, the incidence of adverse effects was significantly lower when morphine was administered alone.
